# Clinical outcome and risk factors for recurrence in borderline ovarian tumours

**DOI:** 10.1038/sj.bjc.6603139

**Published:** 2006-05-09

**Authors:** Y Yokoyama, T Moriya, T Takano, T Shoji, O Takahashi, K Nakahara, H Yamada, N Yaegashi, K Okamura, T Izutsu, T Sugiyama, T Tanaka, H Kurachi, A Sato, T Tase, H Mizunuma

**Affiliations:** 1 Department of Obstetrics and Gynecology, Hirosaki University School of Medicine, 5 Zaifu-cho, Hirosaki 036-8562, Japan; 2 Department of Pathology, Tohoku University School of Medicine, 1-1 Seiryo-cho, Aoba-ku, Sendai 980-8574, Japan; 3 Department of Obstetrics and Gynecology, Tohoku University School of Medicine, 1-1 Seiryo-cho, Aoba-ku, Sendai 980-8574, Japan; 4 Department of Obstetrics and Gynecology, Iwate Medical University School of Medicine, 19-1 Uchimaru, Morioka 020-8505, Japan; 5 Department of Obstetrics and Gynecology, Akita University School of Medicine, 1-1-1 Hondo, Akita 010-8543, Japan; 6 Department of Obstetrics and Gynecology, Yamagata University School of Medicine, 2-2-2 Iidanishi, Yamagata 990-9585, Japan; 7 Department of Obstetrics and Gynecology, School of Medicine Fukushima Medical University, 1 Hikarigaoka, Fukushima 960-1295, Japan; 8 Department of Gynecology, Miyagi Cancer Center, 47-1 Aishima, Natori 981-1293, Japan

**Keywords:** borderline ovarian tumour, conservative surgery, cystectomy, serous tumour, multivariate analysis

## Abstract

We investigated the long-term prognosis of borderline ovarian tumours and determined risk factors for recurrence. One hundred and twenty-one borderline ovarian tumours treated between 1994 and 2003 at the participating institutions in the Tohoku Gynecologic Cancer Unit were retrospectively investigated for clinical stage, histopathological subtype, surgical technique, postoperative chemotherapy, the presence or absence of recurrence, and prognosis. The median follow-up period was 57 months (1–126 months). One hundred and nine cases (90.6%) were at clinical stage I. The histopathological subtypes consisted of 91 cases of mucinous tumour (75.2%), 27 cases of serous tumour (22.3%), and three cases of endometrioid tumour. Conservative surgery was used in 53 cases (43.8%), radical surgery in 68 cases (56.2%), a staging laparotomy in 43 cases (35.5%), and postoperative adjuvant therapy in 30 cases (24.8%). Recurrence was found in eight cases, but no tumour-related deaths were reported. Although no significant difference in disease-free survival rate was seen between different clinical stages, the difference in disease-free survival rate between serous and nonserous (mucinous and endometrioid) types was significant (*P*<0.05). The 10-year disease-free survival rate was 89.1% for the radical surgery group and 57.4% for the conservative surgery group – this difference was significant (*P*<0.05). In the conservative surgery group, cystectomy and serous tumour were independent risk factors for recurrence. Although recurrence was observed, the long-term prognosis of borderline ovarian tumour was favourable, without tumour-related deaths. Considering the favourable prognosis, conservative surgery can be chosen as far as the patient has a nonserous tumour and receive adnexectomy. However, in cases of serous type and/or receiving cystectomy special care should be given as relative risk rates of recurrence elevate by 2–4-folds.

Taylor (1929) found that some epithelial ovarian tumours showed clinically intermediate behaviour between benign and malignant, and called them ‘semimalignant’. The International Federation of Gynecology and Obstetrics (FIGO) has formally introduced this concept as ‘carcinoma of low malignant potential’ in 1971, and the World Health Organization (WHO) as ‘borderline tumour’ in 1973, when the histological diagnostic criteria was proposed. The concept of borderline ovarian tumours was histologically defined as a disease entity that had been proposed clinically, and the adequacy of this histological definition has been repeatedly verified clinically.

With an accumulated experience and knowledge regarding the characteristics and management of borderline ovarian tumours, reclassification and redefinition have been attempted (Seidman and Kurman, 1996), and new prognostic factors have been proposed (de Nictolis *et al*, 1992; Gershenson *et al*, 1999). At present, many conflicting reports are causing confusion. As many of the patients are relatively young (Harris *et al*, 1992), preservation of fertility has been attempted with favourable results (Morice *et al*, 2001). However, there are also reports of recurrence or poor prognosis (Kaern *et al*, 1993; Gilks, 2003), and more precise prognostic factors are required. We believe that it is important to get a clear picture of the present status of borderline ovarian tumours, as it has been more than 30 years since the introduction of the concept of these tumours. Our retrospective multicentre study conducted an overall clinical analysis of borderline ovarian tumours. Our ultimate aim is to investigate the long-term prognosis of borderline ovarian tumours, and to determine the risk factors for recurrence.

## MATERIALS AND METHODS

Information on 124 patients with a diagnosis of epithelial borderline ovarian tumour who were treated at the Tohoku Gynecologic Cancer Unit consisting of eight institutes from 1994 to 2003 was collected using each institutional databases.

Central pathological review was adopted in this study. One of the authors reviewed 124 cases diagnosed by the gynaecological pathologists of each institutes concerning histologic typing and grading of the primary lesion and 121 cases of those were determined as an epithelial borderline tumour. The histopathologic criteria embodied in a recent conference with published commentaries (Bell *et al*, 2004; Ronnett *et al*, 2004), some of which are included in the current WHO classification of ovarian tumours (Tavassoli and Devilee, 2003) were used for the diagnosis of borderline ovarian tumours in this study. These tumours were staged according to FIGO criteria (1987).

Radical surgery was defined as hysterectomy with bilateral salpingo-oophorectomy. Conservative surgery was defined as any surgery that preserved the uterus and one or both ovaries. Conservative surgical procedure was performed as cystectomy or adnexectomy. Peritoneal cytology was performed systematically in both surgical procedures. Surgical staging in the present study was defined as including peritoneal cytology, omentectomy, and pelvic lymphadenectomy with or without paraaortic exploration (lymphadenectomy or biopsy or palpation), and peritoneal biopsy in radical or conservative surgery on occasion. These surgical procedures were performed depending on the surgical teams who provided the treatment and whether borderline tumour was diagnosed during or after the surgical procedure.

With regard to adjuvant chemotherapy, all women with advanced disease, those with stage Ic, and those with a likely persistence of residual tumour after cystectomy received platinum-based treatment in the early years of this study. Thereafter, chemotherapy was usually confined to women with advanced disease.

Comparisons of categorical variables were conduced by two-tailed *χ*^2^ and Fisher's exact tests where appropriate. Evaluation of independent factors predicting disease-specific recurrence was conducted by nominal logistic regression analysis. Survival estimates were calculated using the Kaplan–Meier product limit method. Comparison between survival curves was made using the generalised Wilcoxon's test. Statistical significance was set at *P*<0.05. The patients who lost to follow-up were censored from the survival data.

Detailed information regarding patient's characteristics, treatment method, recurrence, and prognosis of the disease was abstracted from the medical record. We did not request institutional review board approval for this study because of its retrospective nature.

## RESULTS

The median age of patients was 43 years old (range 15–76 years). Fifty-one patients (42.1%) were below 40 years of age, and 29 patients (24.0%) were above 60 years of age (Table 1). The follow-up period varied from 1 to 126 months, with a median of 57 months. One hundred and nine patients (90.6%) had stage I disease, two had stage II disease, and nine had stage III and IV disease (Table 1). The dominant histopathological subtypes were mucinous (91 cases; 75.2%) and serous (27 cases; 22.3%) (Table 1). Only three tumours (2.5%) were of endometrioid type. Seventy-five (82.4%) and 16 of the 91 mucinous borderline tumours were intestinal and endocervical types, respectively. Radical treatment was performed in 68 (56.2%) patients, and 53 (43.8%) patients underwent conservative management (Table 1). Complete surgical staging was performed in 43 (35.5%) patients (Table 1). Adjuvant chemotherapy was given to 30 (24.8%) patients (Table 1). Seventeen patients were lost to follow-up, and two patients died of the other diseases (Table 1). Four patients had a mucinous tumour with pseudomyxoma peritonei and were excluded from the present study because presence of pseudomyxoma peritonei changes the scope of management and the category of pseudomyxoma peritonei is recognised as tumour that can simulate primary mucinous borderline ovarian tumour (Ronnett *et al*, 2004).

**Figure 1:**
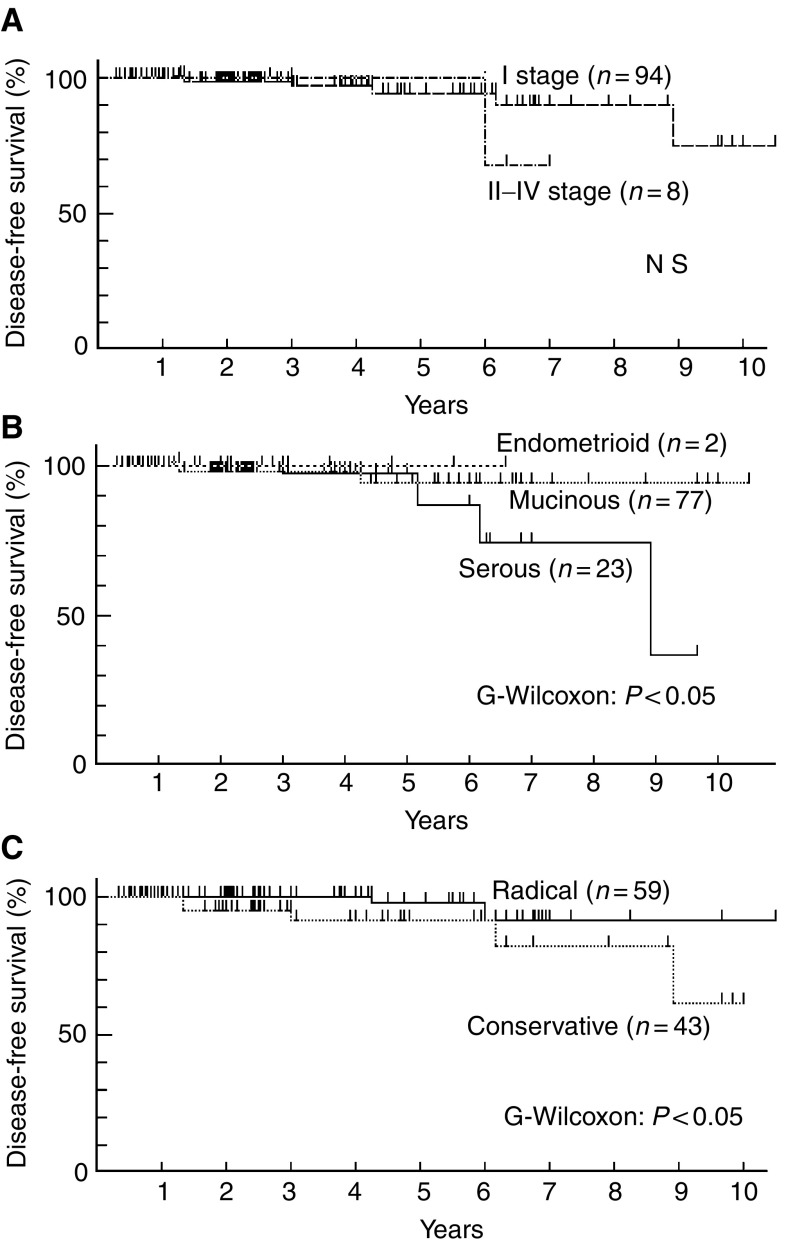
(**A**) Clinical stages and disease-free survival in patients with borderline ovarian tumour. There is no significant difference between two curves. (**B**) Histological type and disease-free survival in patients with borderline ovarian tumour. There is significant difference in disease-free survival between serous and nonserous (mucinous and endometrioid) type (*P*<0.05). (**C**) Surgical procedure and disease-free survival in patients with borderline ovarian tumour. There is significant difference between two curves (*P*<0.05).

Among 102 patients who were finally evaluated for clinical outcome and prognostic factors, eight had tumour recurrence but none of them died of the disease (Table 1). The median time to recurrence was 46±33 months (range 14–107 months). The 5- and 10-year disease-free survival rates were 91.7 and 69.2% for stage I diseases, respectively, and the 5- and 7-year disease-free survival rates were 100 and 66.7% for stage II–IV diseases, respectively (Figure 1A). The 10-year disease-free survival rate was 91.5 and 36.0% for mucinous and serous tumours, respectively (Figure 1B). Although no significant differences in disease-free survival rate were seen between different clinical stages, the difference between serous and nonserous (mucinous and endometrioid) types was significant. On the other hand, the 10-year disease-free survival rate was 89.1% for the radical surgery group and 57.4% for the conservative surgery group (Figure 1C). This difference was significant (*P*<0.05). In univariate analysis, serous type and conservative surgery were found to be important variables affecting recurrence of disease (Table 2). Frequency of recurrence was not influenced by clinical stage, staging laparotomy, and postoperative adjuvant chemotherapy (Table 2). Multivariate analysis showed that only conservative surgery had independent prognostic value regarding recurrence of disease (Hazard ratio 2.2, 95% confidence interval, 0.02–0.52) (Table 2). Subsequently, risk factors for recurrence were evaluated among 43 patients who underwent conservative surgery (Table 3). Of these patients, six had tumour recurrence (Table 3). Three of eight patients who had cystectomy and three of 35 patients who had adnexectomy experienced tumour recurrence (Table 3, *P*<0.03). Recurrence occurred more frequently in patients with serous tumour than with nonserous tumour (Table 3). No correlation was found between recurrence and the factors such as clinical stage, staging laparotomy, or postoperative adjuvant chemotherapy among conservative surgery group (Table 3). Multivariate analysis confirmed cystectomy and serous type as an independent risk factor for recurrence of disease among the patients who underwent conservative surgery (Table 3). Table 4 shows estimated relative risk of having recurrence of disease for different combination of procedure of conservative surgery and histopathological subtype. For example, the relative risk for a patient receiving cystectomy for her serous tumour is 4.33 times greater than the risk for a patient receiving adnectomy for her nonserous tumour.

The clinical and pathological features of the eight patients who developed recurrence were demonstrated in Table 5. None of these eight patients died of progression of their disease. Three of the four serous tumours with recurrence were a noninvasive peritoneal implant, one of which was diagnosed as a serous adenocarcinoma at recurrence. The case developed adenocarcinoma in contralateral ovary 107 months after cystectomy. All mucinous tumours with recurrence were of intestinal subtype. All patients with recurrence who were initially treated conservatively are free of disease after secondary surgical treatment.

## DISCUSSION

It has been shown that the 5-year survival rate was 95–97% for stage I and 65–87% for stages II and III (Trope *et al*, 2000; Trimble *et al*, 2002; Sherman *et al*, 2004) suggesting that the prognosis for borderline ovarian tumours depends on extraovarian extension of the tumour. In addition, prognostic factors included clinical stage, histopathological subtype, and residual tumour, but the surgical method was not regarded as a prognostic factor (Trope *et al*, 2000; Gilks, 2003). The results of the present study, however, showed neither the stage nor the histopathological subtype of the disease was related with long-term prognosis, but showed that disease-free survival rates were significantly lower in cases managed by conservative surgery (Figure 1).

In our study, surgical procedure was found to be an independent risk factor for recurrence and the risk could be reduced by radical surgery (Table 2). Because borderline tumours are seen more frequently in younger females than definitive carcinomas (Harris *et al*, 1992), whether conservative surgery is appropriate for borderline ovarian tumours is an important matter to be resolved. Zanetta *et al* (2001) reported that only three of 119 stage I (2.5%) cases that underwent radical surgery recurred, whereas 20 out of 164 stage I (12.1%) cases that underwent conservative surgery recurred, with one case resulted in death from the disease. Morice *et al* (2001) demonstrated that the majority of recurrent cases, including stages II and III, were cured completely by subsequent surgery, and few cases resulted in death. More over, Donnez *et al* (2003) reported that although recurrence was commoner in cases treated by conservative surgery (3 out of 16, 18.7%) than by radical surgery (0 out of 59, 0%), subsequent treatment resulted in no tumour-related deaths, and 63.6% of conservative surgery cases subsequently became pregnant, suggesting that conservative surgery can be an option for management of borderline malignant ovarian tumours in young subjects who need to reserve fertility. However, it is also reported that all of deaths as a result from recurrence were seen in cases treated by conservative surgery (Morris *et al*, 2000; Zanetta *et al*, 2001). Therefore, it is of quite importance to investigate underlying risk factors for recurrence after conservative surgery. As shown in Table 3, we found that cystectomy and serous tumours were independent risk factors for recurrence in patients who received conservative surgery. Previous reports have shown that recurrence after cystectomy did not necessarily occur ipsilaterally (Morris *et al*, 2000, 2001; Zanetta *et al*, 2001). So it seems that the residual tumour during cystectomy is solely responsible for recurrence. Then, it may be rational for young women who wish pregnancy to select cystectomy as an option if the surgical margin is free of tumour. However, results by Morice *et al* (2001) did not support this as they found that the recurrence rate was high after cystectomy compared with adnexectomy. Morris *et al* (2000) also demonstrated that recurrence was higher in cases treated by cystectomy rather than by adnexectomy. The present study confirmed this and further demonstrated for the first time that a difference in pathohistology affects the recurrence rate. As shown in Table 3, it was revealed that serous tumour is a significant risk factor for recurrence in cases managed by conservative surgery. Morris *et al* (2000) also showed similar tendency, but they regrettably missed statistical analysis. As shown in Table 4, the present study clearly demonstrated that the risk of recurrence when serous tumours were treated by cystectomy was approximately four times higher than for adnexectomy of nonserous tumours.

As a prognostic factor for borderline ovarian serous tumours, the concept of peritoneal implant is attracting attention (Bell *et al*, 1988). When estimating the prognosis of borderline ovarian serous tumours, peritoneal lesions should be explored and biopsied at the time of the surgery – in other words, accurate surgical staging is required. Clinical stage is one of the most important prognostic factors in borderline ovarian tumours (Kliman *et al*, 1986), and an accurate surgical staging is indispensable for follow-up after conservative surgery, as well as selecting postoperative therapy. Winter *et al* (2002) compared 48 cases that underwent complete surgical staging, and 45 cases without surgical staging – a higher stage was found in 17% (8 out of 48) of those assessed by surgical staging, but there was no difference in recurrence and survival rates between the groups. Camatte *et al* (2002) found metastasis to lymph nodes in 19% (8 out of 42) of cases. All cases with metastases were seen with borderline ovarian serous tumours associated with peritoneal dissemination, but no cases resulted in death – there was no difference in prognosis when compared with cases without metastases. The presence or absence of a peritoneal lesion is an important predictive factor of recurrence as well as an important prognostic factor, and we do not deny the importance of surgical examination of the abdominal cavity where possible. However, many reports have indicated that the presence or absence of lymph node metastasis is not related to the prognosis for borderline ovarian tumours (Camatte *et al*, 2002; Winter *et al*, 2002), and it is still debatable whether or not to perform a biopsy or dissection of the lymph nodes. As shown in Table 3, the present study could not show a significant relevance to risk of recurrence. The limitation of the present study is that surgical staging was not considered beforehand in all cases so that our data may be biased in this respect. Further studies using a prospective design with emphasis on surgical staging are required to investigate the risk of recurrence in borderline ovarian serous tumours after conservative surgery. Therefore, it is important that conservative surgery should only be performed in cases that truly require conservative surgery, after giving a full explanation of the risk of recurrence.

Barakat *et al* (1995) reported that cisplatin-based chemotherapy induced complete remission in six of 23 (26%) advanced cases with macroscopic diseases, and in 17 of 25 (68%) cases with microscopic disease, and proposed that adjuvant chemotherapy could be considered as a therapeutic option although a life-extension effect of chemotherapy was not clear. In the present study, the regimen or frequency of chemotherapy used was not uniform, and differed among institutions, and no relationship was found between the presence or absence of postoperative adjuvant chemotherapy and recurrence (Table 2). Kaern *et al* (1993) showed that adjuvant chemotherapy did not improve neither recurrence free survival nor overall survival rate in 364 cases without residual tumour. Morice *et al* (2001) demonstrated that postoperative chemotherapy did not improve the survival rate in 80 cases of advanced borderline ovarian serous tumour in stages II and III with extraovarian extension, and that deaths were more closely related to the treatment than to the tumour. Thus, the efficacy of chemotherapy for borderline ovarian tumours is not yet established.

In conclusion, although recurrence was detected in eight out of 102 cases with borderline ovarian tumour that were available for follow-up, no tumour-related deaths were found, and there was a favourable long-term prognosis. Although the relative risk of recurrence is high, conservative surgery appears to be worth trying to preserve fertility, considering the favourable prognosis. When considering conservative surgery, special care should be given when cystectomy is chosen as a surgical procedure or the histological subtype is borderline serous ovarian tumour. Consensus has not been reached on such issues as to the significance of surgical staging, the indication for postoperative adjuvant chemotherapy, or the indications for conservative surgery. To reinforce the present study results, we expect that a large scaled prospective clinical study involving many institutions will be designed to obtain more evidence.

## Figures and Tables

**Table 1:** Clinical and histopathologic characteristics of patients with borderline tumours

	**Alive with NED**	**Recurrence**	**Died of disease**	**Died of ICD**	**Lost**	**Total**
Number of patients	94	8	0	2	17	121
*Age (years)*						
<20	7	0	0	0	0	7
20–30	14	1	0	0	4	19
31–40	19	4	0	0	2	25
41–50	14	1	0	0	2	17
51–60	17	1	0	1	5	24
> 60	23	1	0	1	4	29
						
*Histological type*						
Mucinous	73	4	0	2	12	91
Serous	19	4	0	0	4	27
Endometriod	2	0	0	0	1	3
						
*Stage*						
Ia	62	4	0	2	10	78
Ib	1	0	0	0	0	1
Ic	24	3	0	0	3	30
II	2	0	0	0	0	2
IIIa	0	1	0	0	1	2
IIIb	1	0	0	0	1	2
IIIc	3	0	0	0	1	4
IV	1	0	0	0	0	1
Unknown	0	0	0	0	1	1
						
*Surgical procedure*						
Radical	57	2	0	2	7	68
Conservative	37	6	0	0	10	53
						
*Staging laparotomy*						
Staged	36	3	0	1	3	43
Unstaged	58	5	0	1	14	78
						
*Adjuvant chemotherapy*						
Yes	22	4	0	1	3	30
No	72	4	0	1	14	91

NED=no evidence of disease; ICD=intercurrent disease.

**Table 2:** Risk factors for recurrence in borderline tumours

	**Recurrence**	**No recurrence**	**Univariate**	**Multivariate**
**Factors**	**(*n*=8)**	**(*n*=94)**	***P***	***P***
Mean age (years)	42.2±13.7	43.5±16.2		
*Histology, n (%)*				
Serous	4 (50)	19 (20.2)		
Nonserous	4 (50)	75 (79.8)	0.053	0.09
				
*Surgical procedure, n (%)*				
Radical	2 (33.3)	57 (60.6)		
Conservative	6 (66.7)	37 (39.4)	0.05	0.031
				
*Staging laparotomy, n (%)*				
Staged	3 (37.5)	36 (38.3)		
Unstaged	5 (62.5)	58 (61.7)	0.96	0.58
				
*Stage, n (%)*				
I	7 (87.5)	87 (92.6)		
II–IV	1 (12.5)	7 (7.4)	0.61	0.79
				
*Adjuvant chemotherapy, n (%)*				
Yes	4 (50)	22 (23.4)		
No	4 (50)	72 (76.6)	0.098	0.33

**Table 3:** Risk factors for recurrence in the patients who underwent conservative surgery for borderline tumours

	**Recurrence**	**No recurrence**	**Univariate**	**Multivariate**
**Factors**	**(*n*=6)**	**(*n*=37)**	***P***	***P***
*Surgical procedure, n (%)*				
Cystectomy	3 (50)	5 (13.5)		
Adnexectomy	3 (50)	32 (86.5)	0.03	0.047
				
*Staging laparotomy, n (%)*				
Staged	1 (16.7)	3 (8.1)		
Unstaged	5 (83.3)	34 (91.9)	0.51	0.137
				
*Adjuvant chemotherapy, n (%)*				
Yes	3 (50)	7 (18.9)		
No	3 (50)	30 (81.1)	0.095	0.593
				
*Stage, n (%)*				
Ia	3 (50)	23 (62.2)		
Ic	3 (50)	14 (37.8)	0.57	0.198
				
*Histology, n (%)*				
Serous	3 (50)	6 (16.2)		
Non-serous	3 (50)	31 (83.8)	0.059	0.041

**Table 4:** Relative risk of recurrence in borderline tumours

	**Histological type**	
**Conservative surgery**	**Nonserous**	**Serous**
Adnexectomy	1	2.11
Cystectomy	2.05	4.33

**Table 5:** Eight patients with borderline tumour who developed recurrence

**Age**	**Histological type**	**Stage**	**Initial surgery**	**Staging procedure**	**Adjuvant chemotherapy**	**Time to recurrence**	**Site of recurrence**	**Treatment after recurrence**	**Histology of recurrence site**	**Status**
33	SBT, noninvasive	Ia	Conservative (adnexectomy)	(−)	(+)	74	Intrapelvis	Surgery alone	SBT	NED
35	SBT, noninvasive	Ia	Conservative (adnexectomy)	(−)	(−)	36	Contralateral ovary	Surgery alone	SBT	NED
36	SBT, noninvasive	Ia	Conservative (cystectomy)	(−)	(+)	107	Contralateral ovary	Surgery+chemotherapy	Serous adenocarcinoma	NED
46	SBT, invasive	IIIa	Radical	(+)	(+)	62	Perihepatic	Chemotherapy alone	Unknown	AWD
28	MBT, intestinal	Ic	Conservative (cystectomy)	(−)	(−)	14	Ipsilateral ovary	Surgery alone	MBT, intestinal	NED
35	MBT, intestinal	Ic	Conservative (cystectomy)	(+)	(+)	16	Intrapelvis	Surgery alone	MBT, intestinal	NED
58	MBT, intestinal	Ic	Conservative (adnexectomy)	(−)	(−)	20	Intrapelvis	Surgery alone	MBT, intestinal	NED
67	MBT, intestinal	Ia	Radical	(+)	(−)	35	Lung	None	Unknown	AWD

SBT=serous borderline tumour; MBT=mucinous borderline tumour; NED=no evidence of disease; AWD=alive with disease.
